# Evidential Estimation of an Uncertain Mixed Exponential Distribution under Progressive Censoring

**DOI:** 10.3390/e22101106

**Published:** 2020-09-30

**Authors:** Kuang Zhou, Yimin Shi

**Affiliations:** School of Mathematics and Statistics, Northwestern Polytechnical University, Xi’an 710072, Shaanxi, China; ymshi@nwpu.edu.cn

**Keywords:** uncertain mixed exponential distribution, evidential likelihood, belief function theory, progressive censoring

## Abstract

In this paper, the evidential estimation method for the parameters of the mixed exponential distribution is considered when a sample is obtained from Type-II progressively censored data. Different from the traditional statistical inference methods for censored data from mixture models, here we consider a very general form where there is some uncertain information about the sub-class labels of units. The partially specified label information, as well as the censored data are represented in a united frame by mass functions within the theory of belief functions. Following that, the evidential likelihood function is derived based on the completely observed failures and the uncertain information included in the data. Then, the optimization method using the evidential expectation maximization algorithm (E2M) is introduced. A general form of the maximal likelihood estimates (MLEs) in the sense of the evidential likelihood, named maximal evidential likelihood estimates (MELEs), can be obtained. Finally, some Monte Carlo simulations are conducted. The results show that the proposed estimation method can incorporate more information than traditional EM algorithms, and this confirms the interest in using uncertain labels for the censored data from finite mixture models.

## 1. Background

Mixture models are of great importance in many applied sciences such as survival analysis, pattern recognition, image analysis, economics, and so on [1,2]. In reliability analysis, there are only one population and one type of failure in the simple case of life distributions. However, in real applications, there may be more than one failure cause [3,4]. In this case, the response of the modeling process can be seen as from several distinct sub-populations, and finite mixture models can be used to represent the time of failures.

Suppose the life lengths of these *n* units are independent and identically distributed (i.i.d.) random variables with probability density function (pdf) f(x;θ) and cumulative distribution function (cdf) F(x;θ). Consider the mixture model of the form:

(1) $$f\left(x;\theta \right)\;=\;\sum_{k=1}^{m}\pi_{k}f_{k}\left(x;\lambda_{k}\right),$$

where $\theta =\{\lambda_{1},\;\cdots ,\;\lambda_{m};\pi_{1},\;\cdots ,\;\pi_{m}\}$, with $\pi_{j}\geq 0,j=1,\;\cdots ,\;m$ and $\sum_{j}\pi_{j}=1$. In this paper, we focus on the case where $f_{k}\left(x;\lambda_{k}\right)$ is the exponential density, although the model we develop can be applied more generally. The mixed exponential distribution (MED) model with *m* components has its pdf and cdf respectively as:

(2) $$f\left(x;\theta \right)\;=\;\sum_{k=1}^{m}\pi_{k}f_{k}\left(x;\lambda_{k}\right)=\sum_{k=1}^{m}\pi_{k}\lambda_{k}e^{-\lambda_{k}x}$$

and:

(3) $$F\left(x;\theta \right)\;=\;1-\sum_{k=1}^{m}\pi_{k}s_{k}\left(x;\lambda_{k}\right)=1-\sum_{k=1}^{m}\pi_{k}e^{-\lambda_{k}x},$$

where the parameters are $\pi =(\pi_{1},\;\cdots ,\;\pi_{m})$ and $\lambda =(\lambda_{1},\;\cdots ,\;\lambda_{m})$ with:

(4) $$\sum_{k=1}^{m}\pi_{k}=1,\;0<\pi_{k}<1,$$

and:

$$\lambda_{k}>0,\;k=1,2,\;\cdots ,\;m.$$

As we can see, the samples from mixed distributions are not precisely labeled by their origin sub-classes; thus, the heterogeneous dataset cannot be explicitly decomposed to homogeneous subgroups. Consequently, it brings about a barrier for estimating finite mixture models [5].

Consider a life-testing experiment where *n* units are placed under observation. For some reason, such as to save time and cost, we have to terminate the experiment before all items have failed. In real applications, the removal of units prior to failure is often pre-planned. Data obtained from such a type of experiments are called censored data. The most common censoring schemes are termed Type-I and Type-II censoring. In Type-I censoring, the experiment continues up to a prescribed time *T*. Any failures that occur after *T* cannot been observed. The endpoint of the experiment *T* is assumed to be independent of the failure times. In Type-II censoring, the experiment is terminated upon the $N_{th}$ failure, where N<n is prefixed. The experiments under these two kinds of test schemes have the drawback that they do not allow removal of samples at time points other than the termination of the experiment. However, the Type-II progressive censoring, which can be seen as a generalization of Type-II censoring, allows removing some fixed number of units at the time of the first observed m−1 failures and removing all the remaining at the time of the $m_{th}$ failure, at which time, the experiment terminates [6]. As a result, it is efficient in time and money [7] and has become very popular in the last few years [8,9,10,11,12,13,14].

The parameter estimation problems for different lifetime distributions, including the mixed exponential distribution and some other mixed distribution models, have been widely studied under different censoring schemes [15,16,17]. The most commonly adopted methods to obtain the estimations for mixture models are simply using the expectation maximization (EM) algorithm or Bayesian method. Lee and Scott [18] presented the EM algorithm for fitting multivariate Gaussian mixture models to data that are truncated, censored, or truncated and censored. In [19,20], the authors discussed the parameter estimation methods of the MED under Type-II progressively censored data and progressively hybrid Type-II censored data, respectively. Tahir et al. [21] studied the problem of estimating the parameters of a three-component mixture of exponential, Rayleigh and Burr Type-XII distributions using the Type-I right censoring scheme in the Bayesian framework. The maximum likelihood and Bayesian estimators of the parameters of a heterogeneous population represented by a finite mixture of two Pareto distributions were discussed in [22]. Feroze and Aslam [23] introduced the Bayesian approach for estimating the parameters of the two-component mixture of Weibull distribution under doubly censored samples. Feroze and Aslam [24] considered the Bayesian analysis of the three-component mixture of the Rayleigh distribution under doubly censored samples. All these works assume that we do not have any information about the label of the sub-class at all. However, in many applications, often, it is easy to get some imprecise and uncertain knowledge about the sub-class labels. For instance, in medical surveillance databases, we can find partially labeled data provided by experts or from experience, that is, while not completely unlabeled, there is only uncertain information about class values [5]. The above-mentioned methods cannot deal with the possible available partial label information in the dataset from mixture models.

Zio [25] pointed out that uncertain information is a big challenge in reliability engineering. Specifically, there indeed exits many kinds of uncertain information in reliability analysis for censored data. First, we only know that the failure time of the censored unit belongs to the interval $\left[t^{*},+\infty \right)$. Second, as mentioned, in the finite mixture models, there may be some uncertain information about the sub-class labels of the data. The theory of belief functions (also known as Dempster–Shafer theory (DST)) is appealing to represent data uncertainty. As an extension of probability theory, it has many advantages in dealing with uncertain information. Similar to the probability distribution over the discernment frame, in DST, the basic belief assignment (BBA) defined on the power set of the frame is used to represent the available information. Many scholars have studied how to measure the uncertainty of BBA [26,27,28,29]. Due to the effectiveness of DST in dealing with uncertain knowledge, it has already been widely used in many fields such as data classification/clustering [30,31], target recognition [32], decision making [33], fault diagnosis [34,35], complex networks [36,37], and so on.

In this paper, we try to handle the uncertain information in mixed distributions under Type-II progressive censoring using the theory of belief functions. Note that for the progressively censored data considered in this work, the incomplete observations are not influenced by the specific values taken by the random variables. Thus, the mechanism that causes complete failure data cases to become incompletely reported can be ignored. Under such a kind of coarsening at random (CAR) assumption, the statistical inference can proceed based on the so-called “face-value likelihood”, which measures the probability of incomplete observations by its marginal probability according to the underlying complete data distribution [38,39]. The EM algorithm is quite effective at maximizing the face-value likelihood [38], and it has been widely used for progressively censored data [7,40]. Different from the traditional estimation method using the EM algorithm, the proposed evidential parameter estimation model can take not only the uncertain censored observations, but also the prior partial information about class labels into consideration. The two kinds of uncertain information are modeled in a united frame by mass functions in belief function theory, and then, the evidential likelihood function is derived. The optimization method to get the optimal estimators, called maximal evidential likelihood estimates (MELEs), is derived based on the evidential-EM (E2M) algorithm [41]. Experimental results show that the proposed model can take advantage of the the partial information about the sub-class labels effectively and consequently improve the performance of the estimation model.

The rest of this paper is organized as follows. In Section 2, some basic concepts and the rationale of our method are briefly introduced. In Section 3, the evidential estimation model to get MELEs, which can maximize the evidential likelihood function, is presented in detail. In order to show the effectiveness of our approach, a real data application is discussed in Section 4, while some Monte Carlo simulations are conducted in Section 5. Finally, we conclude the paper in Section 6.

## 2. Preliminary Knowledge

Some necessary background knowledge related to this paper will be recalled in this section. The Type-II progressive censoring scheme is first introduced in Section 2.1. The basic knowledge about the theory of belief functions is then presented in Section 2.2. Finally, the evidential likelihood is recalled in Section 2.3.

### 2.1. Type-II Progressively Censoring Scheme

The Type-II progressive censoring scheme can be described as follows. Suppose *n* independent identical items are placed on a life-test. The integer N(<n), which denotes the number of failures we would like to observe in the experiment, is fixed before the experiment. Let $R=(R_{1},R_{2},\;\cdots ,\;R_{N})$, where $R_{1},R_{2},\;\cdots ,\;R_{N}$ are also fixed integers describing the progressive censoring scheme. They should satisfy:

$$R_{1}+R_{2}+\cdots +R_{N}+N=n.$$

At the time of the first failure, say $T_{1}$, $R_{1}$ of the remaining units are randomly removed. Similarly, at the time of the $i_{th}$ failure, say $T_{i}$, $R_{i}\;\left(i=1,2,\;\cdots ,\;N\right)$ of the remaining units are removed. At the time of the $N_{th}$ failure, say $T_{N}$, the remaining $R_{N}=n-N-\sum_{i=1}^{N-1}R_{i}$ items are removed, and the experiment terminates. Therefore, in the presence of the Type-II progressive censoring scheme, the observed failures are $\left\{T_{1},\;\cdots ,\;T_{N}\right\}$. For further details on this censoring scheme, the readers may refer to the excellent monograph of Balakrishnan and Aggarwala [6].

### 2.2. Theory of Belief Functions

The theory of belief functions is a mathematical theory that generalizes the theory of probabilities by giving up the additivity constraint. In this theory, justified degrees of support are assessed according to an evidential corpus, which is the set of all evidential pieces of evidence held by a source that justifies the degrees of support assigned to some subsets.

Let $\Omega =\{\omega_{1},\omega_{2},\ldots ,\omega_{c}\}$ be the finite domain of reference, called the discernment frame. The *c* elements in Ω are nonempty and mutually exclusive hypotheses related to a given problem. The belief functions are defined on the power set $2^{\Omega }=\left\{A:A\subseteq \Omega \right\}$. The function $m:2^{\Omega }\to \left[0,1\right]$ is said to be the basic belief assignment on $2^{\Omega }$, if it satisfies:

(5) $$\sum_{A\subseteq \Omega }m\left(A\right)=1.$$

Every $A\in 2^{\Omega }$ such that m(A)>0 is called a focal element. The difference from probability models is that masses can be given to any subset of Ω instead of only to the atomic element of Ω. The credibility and plausibility functions are defined as in Equations (6) and (7), respectively:

(6) $$Bel(A)=\sum_{B\subseteq A,B\neq \emptyset }m(B),\;\;\forall A\subseteq \Omega ,$$

(7) $$Pl(A)=\sum_{B\cap A\neq \emptyset }m(B),\;\;\forall A\subseteq \Omega .$$

Each quantity Bel(A) measures the total support given to *A*, while Pl(A) represents the potential amount of support to *A*. The function pl:Ω→[0,1] such that pl(ω)=Pl({ω}) is called the contour function associated with *m*.

According to the type of the focal elements, we can define some particular mass functions. A categorical mass function is a normalized mass function that has a unique focal element $A^{*}$. This kind of mass function can be defined as:

(8) $$m\left(A\right)=\left\{\begin{array}{cc} 1 & \mathrm{if}\;\;A=A^{*}\subset \Omega \\ 0 & \mathrm{otherwise}. \end{array}\right.$$

A vacuous mass function is a particular categorical mass function focused on Ω. It is a special kind of categorical mass function with a unique focal element Ω. This type of mass function is defined as follows:

(9) $$m\left(A\right)=\left\{\begin{array}{cc} 1 & \mathrm{if}\;\;A=\Omega \\ 0 & \mathrm{otherwise}. \end{array}\right.$$

The vacuous mass function emphasizes the case of total ignorance.

A Bayesian mass function is a mass function for which all focal elements are elementary hypotheses, i.e., the focal elements are all singletons. It is defined as follows:   

(10) $$m\left(A\right)=\left\{\begin{array}{cc} a\in [0,1] & \mathrm{if}\;\;|A|=1\\ 0 & \mathrm{otherwise}. \end{array}\right.$$

As all focal elements are single points, this mass function is a probability distribution over frame Ω. Specifically, if a Bayesian mass function is categorical, it describes that there is no uncertainty at all, and we are completely sure about the state of the variable concerned.

If $m_{1}$ and $m_{2}$ are two independent mass functions defined on Ω, a new mass function can be formed by combining $m_{1}$ and $m_{2}$ using Dempster’s rule directly:

(11) $$\left(m_{1}⊕m_{2}\right)\left(A\right)\;=\;\frac{1}{1-k}\sum_{B\cap C=A}m_{1}\left(B\right)m_{2}\left(C\right),$$

where *k* is defined as

(12) $$k\;=\;\sum_{B\cap C=\emptyset }m_{1}\left(B\right)m_{2}\left(C\right).$$

This describes the conflict between $m_{1}$ and $m_{2}$. When k=1, the two masses are completely in conflict, and they cannot be combined using Dempster’s rule.

Suppose $m_{1}$ is a Bayesian mass function, and its corresponding contour function is a probability distribution function defined by $p_{1}\left(\omega \right)=m\left(\left\{\omega \right\}\right)$. Assume $m_{2}$ is an arbitrary mass function with contour function $pl_{2}\left(\omega \right)$. The fused mass function of $m_{1}$ and $m_{2}$ by Dempster’s rule yields a Bayesian mass function. Its corresponding contour function can be defined by [41]:

(13) $$\left(p_{1}⊕pl_{2}\right)\left(\omega \right)\;=\;\frac{1}{1-k}\;p_{1}\left(\omega \right)pl_{2}\left(\omega \right),$$

where *k* is the conflict between $m_{1}$ and $m_{2}$. It can be written as:

(14) $$k=1-\sum_{w\in \Omega }p_{1}\left(\omega \right)pl_{1}\left(\omega \right).$$

The item $\sum_{w\in \Omega }p_{1}\left(\omega \right)pl_{1}\left(\omega \right)$ can be regarded as the mathematical expectation of $pl_{2}$ with respect to $p_{1}$. If $m_{2}$ is categorical and such that $m_{2}\left(A\right)=1$, then $p_{1}⊕pl_{2}$ is the probability distribution by conditioning $p_{1}$ with respect to *A*.

Let $m^{X}$ and $m^{Y}$ be two mass functions defined on $\Omega_{X}$ and $\Omega_{Y}$, respectively, and $Pl^{X}$ and $Pl^{Y}$ are the associated plausibility functions. $Pl^{XY}$ is the plausibility function defined on the product frame $\Omega_{X}\times \Omega_{Y}$. Variables *X* and *Y* are called cognitively independent if:

(15) $$Pl^{XY}\left(A\times B\right)=Pl^{X}\left(A\right)Pl^{Y}\left(B\right),\forall A\subseteq \Omega_{X},B\subseteq \Omega_{Y}.$$

If two variables are cognitively independent, the evidence on one variable does not affect the beliefs on other variables. It is clear that cognitive independence reduces to stochastic independence when $m^{X}$ and $m^{Y}$ are Bayesian.

### 2.3. Evidential Likelihood

Let *X* be a random vector with probability density function $p_{X}\left(\cdot ;\theta \right)$, where θ is an unknown parameter. Assume that *X* is perfectly observed, and let $x_{0}$ be a realization of *X*. The complete likelihood function given $x_{0}$ would be defined as:

(16) $$L\left(\theta ;x_{0}\right)\;=\;p\left(x_{0};\theta \right),\;\;\forall \theta \in \Theta .$$

If *x* is not precisely observed, but we only know that x∈A for $A\subseteq \Omega_{X}$, where $\Omega_{X}$ is the set from which *X* can take values. Then, the likelihood function given such imprecise data can be defined as [41]:   

(17) $$L\left(\theta ;A\right)\;=\;\sum_{x\in A}p\left(x;\theta \right),\;\;\forall \theta \in \Theta .$$

More generally, if the observation *x* is not only imprecise, but also uncertain, it can be described by a mass function *m* on $\Omega_{X}$ with focal elements $A_{1},\;\cdots ,\;A_{r}$. The corresponding masses assigned to each focal element are $m\left(A_{1}\right),\ldots ,m\left(A_{r}\right)$. The likelihood function given such uncertain data can be extended as [41]:

(18) $$L\left(\theta ;m\right)\;=\;\sum_{i=1}^{r}m\left(A_{i}\right)L\left(\theta ;A_{i}\right).$$

It can be rewritten as:

(19a)L(θ;m)=∑i=1rm(Ai)∑x∈Aip(x;θ)(19b)=∑x∈ΩXp(x;θ)∑Ai∋x,Ai⊆ΩXm(Ai)(19c)=∑x∈ΩXp(x;θ)pl(x)(19d)=Eθ[pl(X)](19e)≜L(θ;pl).

From Equation (19c), we can see that L(θ;m) depends on *m* through its associated contour function pl. As a result, L(θ;m) can be denoted by L(θ;pl) indifferently. We can see that L(θ;pl) is the expectation of pl(x) with respect to p(x;θ). It is often called the evidential likelihood function [42].

The evidential likelihood function L(θ;pl) can be seen as a special case of L(θ;A) and L(θ;x). If pl is associated with a categorical mass function $m_{A}$, then:

$$pl\left(x\right)=\left\{\begin{array}{cc} 1, & x\in A\subseteq \Omega_{X},\\ 0, & \mathrm{otherwise}. \end{array}\right.$$

Equation (19c) equals the likelihood function given the imprecise data in Equation (17). If pl is associated with a certain mass function:

$$pl\left(x\right)=\left\{\begin{array}{cc} 1, & X=x_{0},\\ 0, & \mathrm{otherwise}. \end{array}\right.$$

Equation (19c) degrades to the complete likelihood defined in Equation (16).

## 3. Maximum Evidential Likelihood Estimates for Uncertain Progressively Censored Data

The EM algorithm is usually adopted to obtain the maximal likelihood estimates (MLEs) for the datasets with missing information. However, it cannot deal with the partially labeled information included in the finite mixture model. In this section, we discuss the evidential likelihood for the uncertain progressively censored data from mixture distributions. The optimal statistical estimates that can be obtained by maximizing the evidential likelihood function (named MELEs) are derived, and the relation with the traditional EM model is also discussed.

### 3.1. Evidential Likelihood for Uncertain Progressively Censored Data

Assume that $X_{1},X_{2},\ldots ,X_{n}$ are *n* independent variables that follow the mixture distribution in the form of Equation (1). Denote their probability density function by f(x;π,λ), the probability distribution function by F(x;π,λ) and the survival function by s(x;π,λ). Suppose *n* units are placed on a life-test under the progressive censoring scheme.

In the Type-II progressive censoring scheme, we can denote the observed failure times by $T_{i},i=1,2,\ldots ,N$, and use $T=(t_{1},t_{2},\ldots ,t_{N})$ to describe the completely observed data. At the time of the $i_{th}$ observed failure, we remove $R_{i}\left(i=1,2,\;\cdots ,\;N\right)$ items. Denote the failure times of these units by:

$$Z=\{z_{ij},i=1,2,\ldots ,N;j=1,2,\ldots ,R_{i}\}.$$

It is easy to know that *Z* cannot be observed in the experiment.

Let $B_{i}^{\left(1\right)}=\left(b_{i1}^{\left(1\right)},b_{i2}^{\left(1\right)},\ldots ,b_{im}^{\left(1\right)}\right)$. Each $b_{ij}^{\left(1\right)}$ in $B_{i}^{\left(1\right)}$ denotes whether the completely observed failure $t_{i}$ comes from the $j_{th}$ component of the mixture model. If $T_{i}∼f_{j}\left(x;\theta \right)$, $b_{ij}^{\left(1\right)}=1$. Otherwise, $b_{ij}^{\left(1\right)}=0$. Similarly, we can define the indicator for the unobserved data $z_{ij}$. Let $B_{ij}^{\left(2\right)}=\left(b_{ij1}^{\left(2\right)},b_{ij2}^{\left(2\right)},\ldots ,b_{ijm}^{\left(2\right)}\right)$, where $b_{ijk}^{\left(2\right)}$ represents whether the $j_{th}$ removed unit at the time $t_{i}$ comes from the $k_{th}$ component in the mixture model. Let $B^{\left(1\right)}=\left\{B_{i}^{\left(1\right)},i=1,2,\ldots ,N\right\}$ and $B^{\left(2\right)}=\left\{B_{ij}^{\left(2\right)},i=1,2,\;\cdots ,\;N,j=1,2,\;\cdots ,\;R_{i}\right\}$.

The complete variables in this life-test can be denoted by W=(X,B), where X=(T,Z) and $B=(B^{\left(1\right)},B^{\left(2\right)})$. It is clear that the observed data in this model are *T*, and the hidden variables are *Z* and *B*. Let θ=(π,λ), then the probability function of *W* under the Type-II progressive censoring scheme is:

(20) $$p\left(w;\theta \right)=\prod_{i=1}^{N}\left[\prod_{k=1}^{m}{\left(\pi_{k}f_{k}\left(t_{i};\lambda_{k}\right)\right)}^{b_{ik}^{\left(1\right)}}\cdot \prod_{j=1}^{R_{i}}\prod_{k=1}^{m}{\left(\pi_{k}f_{k}\left(z_{ij};\lambda_{k}\right)\right)}^{b_{ijk}^{\left(2\right)}}\right].$$

The likelihood function based on the complete data is:

(21) L(θ;w)=p(w;θ),

As mentioned, in this life-test, the observed data are $T=\{t_{i},i=1,2,\;\cdots ,\;N\}$. The likelihood function based on the observed data is:

(22) $$L(\theta )\;=\;C\prod_{i=1}^{N}[f\left(t_{i};\theta \right)\left(s{\left(t_{i};\theta )\right)}^{R_{i}}\right],$$

where *C* is a constant number. The maximum likelihood estimates can be obtained by maximizing Equation (22). The EM algorithm is usually adopted to get the MLEs for the progressively censored data [7]. As we can see, this likelihood function is based on the observed data, but it ignores some possible uncertain information of the partial labels. In fact, there are two kinds of uncertain information in this case. One is the failure time of the removed units during the experiment, for which we only know their censored time. The other is the sub-class label of each unit. Here, we use the mass functions in belief function theory to model these two types of uncertain information in a united frame. In order to establish a united frame for the data, we can describe all the data obtained in the life-test using different kinds of mass functions.

If *X* is a completely observed data and we assume that $X=t^{*}$, we can model this information with a certain mass function. Its contour function can be defined as:

(23) $$pl_{1}^{\left(X\right)}\left(x\right)=\left\{\begin{array}{cc} 1, & x=t^{*},\\ 0, & \mathrm{otherwise}. \end{array}\right.$$

If *X* is the censored data at time $t^{*}$, we only know that $x\in [t^{*},+\infty )$. We can model this uncertain information using the following contour function:

(24) $$pl_{2}^{\left(X\right)}\left(x\right)=\left\{\begin{array}{cc} 1, & x\in [t^{*},+\infty ),\\ 0, & \mathrm{otherwise}. \end{array}\right.$$

As mentioned before, in real applications, some uncertain information could be obtained from the experts or from historical data. The plausibility is adopted to express the uncertain information about the partial class labels of units. For the completely observed data $t_{i}$, the plausibility about the proposition that the unit with failure $t_{i}$ comes from the $k_{th}$ component is $pl_{t_{i},k}^{\left(B\right)}$. For the censored data $z_{ij}$, we can use $pl_{z_{ij},k}^{\left(B\right)}$.

Suppose that the failure time *X* and the sub-class label *B* are cognitively independent. Therefore, the contour function of the complete data $w_{i}=\left(x_{i},b_{i}\right)$ can be defined by:

(25) $$pl\left(w_{i}\right)=\prod_{k=1}^{m}{\left(pl_{x_{i},k}^{\left(B\right)}\right)}^{b_{ik}}\cdot pl^{\left(X\right)}\left(x_{i}\right).$$

where $pl_{x_{i},k}^{\left(B\right)}$ is the plausibility that $x_{i}$ comes from the $k_{th}$ sub-group and $pl^{\left(X\right)}\left(x_{i}\right)$ describes the information about the failures. For the completely observed data and censored data, $pl^{\left(X\right)}\left(x_{i}\right)$ can be defined as Equations (23) and (24), respectively.

Considering the progressive censoring scheme, given the data w=(x,b), the contour function of *x* can be defined as:

(26a)pl(w)=∏i=1npl(X)(xi)pl(B)(bi)(26b)=∏i=1n∏k=1mplxi,k(B)bikp(X)(xi)(26c)=∏i=1N∏k=1mplti,k(B)bik(1)pl1(X)(ti)·∏j=1Ri∏k=1mplzij,k(B)bijk(2)pl2(X)(zij).

The evidential likelihood for the progressively censored data can be derived as:

(27a)L(θ;pl)=∏i=1nEα[pl(Wi)](27b)=∏i=1nEα[pl(X)(Xi)pl(B)(Bi)](27c)=∏i=1n∑k=1mπkEα[pl(X)(Xi)pl(B)(Bi)|bik=1](27d)=∏i=1n∑k=1mπkplxi,k(B)Eα[pl(X)(Xi)|bik=1]=∏i=1N∑k=1mπkplti,k(B)Eα[pl1(X)(ti)|bik(1)=1](27e)·∏i=1N∏j=1Ri∑k=1mπkplzij,k(B)Eα[pl2(X)(zij)|bijk(2)=1](27f)=∏i=1N∑k=1mπkplti,k(B)fk(ti;λk)·∏j=1Ri∑k=1mπkplzij,k(B)sk(ti;λk),

where θ=(π,λ) denotes the unknown parameters.

**Corollary** **1.** *If there is no information about the sub-class label of each unit, we can use the vacuous mass function on $\Omega_{B}$, i.e., $pl_{x_{i},k}^{\left(B\right)}=1$. Therefore, for the observed data and the censored data, we can get:*

(28) $$\begin{array}{cc} pl_{t_{i},k}^{\left(B\right)} & =1,i=1,2,\ldots ,N;k=1,\ldots ,m,\\ pl_{z_{ij},k}^{\left(B\right)} & =1,i=1,2,\ldots ,N;j=1,2,\ldots ,R_{i};k=1,\ldots ,m. \end{array}$$

*In this case, the evidential likelihood function defined in Equation *(27f)* can be rewritten as:*

(29) $$L\left(\theta ;pl\right)\;=\;\prod_{i=1}^{N}\left[\left(\sum_{k=1}^{m}\pi_{k}f_{k}\left(t_{i};\lambda_{k}\right)\right)\cdot {\left(\sum_{k=1}^{m}\pi_{k}s_{k}\left(t_{i};\lambda_{k}\right)\right)}^{R_{i}}\right].$$

*This is the same as Equation *(22)*.*

From Corollary 1, we can see that the traditional likelihood can be regarded as a special case of the evidential likelihood for the progressively censored data when there is no prior information about the sub-class label of each unit.

### 3.2. The Optimal Estimates under the Evidential Likelihood

The E2M algorithm can be evoked to derive the MELEs for the uncertain progressively censored data from mixture models. Given the initial parameter value of $\theta =\theta^{\left(0\right)}$, the E-step and M-step are repeated alternatively.

- E-step: Derive the *Q* function, which can be seen as the expectation of logL(θ;w) with respect to $p(w|pl;\theta^{\left(q\right)})$.
  From Equation (13), the probability mass function $p(\cdot |pl;\theta^{\left(q\right)})$ can be expressed as:
  (30) $$p\left(w|pl;\theta^{\left(q\right)}\right)=\frac{p_{1}\left(w;\theta^{\left(q\right)}\right)pl_{2}\left(w\right)}{\sum_{w^{\prime }\in \Omega }p_{1}\left(w^{\prime };\theta^{\left(q\right)}\right)pl_{2}\left(w^{\prime }\right)}=\frac{p\left(w;\theta^{\left(q\right)}\right)pl\left(w\right)}{L(\theta^{\left(q\right)};pl)}.$$
  The probability function $p(\cdot |pl;\theta^{\left(q\right)})$ can be seen as the Dempster combination of $p(w;\theta^{\left(q\right)})$ and pl(w). The former describes the random uncertainty due to the underlying population, while the latter reflects the epistemic uncertainty due to the partial knowledge.
  According to Equation (30), the *Q* function at the $q_{th}$ iteration is:
  (31a)Q(θ,θ(q))=E[logL(θ;w)|pl;θ(q)](31a)=∑w∈ΩWlog(L(θ;w))p(w;θ(q))pl(w)L(θ(q);pl),
  where L(θ;w) is the likelihood function based on the complete data (see Equation (21)).
- M-step: This is the same as the traditional EM algorithm. In this step, the value of θ can be obtained by maximizing $Q(\theta ,\theta^{\left(q\right)})$.

**Theorem** **1.** *$L(\theta^{\left(q\right)};pl),q=0,1,2,\cdots$ of the evidential likelihood function obtained by the above-mentioned E2M algorithm is not decreasing, i.e., it verifies:*

(32) $$L\left(\theta^{\left(q+1\right)};pl\right)\geq L\left(\theta^{\left(q\right)};pl\right).$$

**Proof.**  From Equation (30), we know:   

(33) $$p\left(w|pl;\theta \right)=\frac{p(w;\theta )pl(w)}{L(\theta ;pl)}=\frac{L(\theta ;w)pl(w)}{L(\theta ;pl)}.$$

From the above equation, we can get:

(34) $$\frac{L(\theta ;w)}{L(\theta ;pl)}=\frac{p(w|pl;\theta )}{pl(w)}≜g\left(w|p;\theta \right).$$

Then, we can have:

(35) logL(θ;pl)=logL(θ;w)−logg(w|pl;θ).

Taking the expectation of both sides of the above equation with respect to $p(w|pl;\theta^{\left(q\right)})$, then it is easy to get:

(36) $$\log L\left(\theta ;pl\right)=E\left[\log L\left(\theta ;w\right)|pl;\theta^{\left(q\right)}\right]-E\left[\log g\left(x|pl;\theta \right)|pl;\theta^{\left(q\right)}\right].$$

Let

(37) $$H\left(\theta ,\theta^{\left(q\right)}\right)=E\left[\log g\left(x|pl;\theta \right)|pl;\theta^{\left(q\right)}\right],$$

and then, we have:

(38) $$\begin{array}{c} \log L\left(\theta^{\left(q+1\right)};pl\right)-\log L\left(\theta^{\left(q\right)};pl\right)\\ =\left(Q\left(\theta^{\left(q+1\right)},\theta^{\left(q\right)}\right)-Q\left(\theta^{\left(q\right)},\theta^{\left(q\right)}\right)\right)-\left(H\left(\theta^{\left(q+1\right)},\theta^{\left(q\right)}\right)-H\left(\theta^{\left(q\right)},\theta^{\left(q\right)}\right)\right). \end{array}$$

It is easy to see that $Q\left(\theta^{\left(q+1\right)},\theta^{\left(q\right)}\right)\geq Q\left(\theta^{\left(q\right)},\theta^{\left(q\right)}\right)$ since in the M-step, the value of *θ* is updated by maximizing $Q(\theta ,\theta^{\left(q\right)})$ with respect to *θ*. For any *θ*,

H(θ,θ(q))−H(θ(q),θ(q))(39a)=Elogg(w|pl;θ)g(w|pl;θ(q))|pl;θ(q)(39b)≤logEg(w|pl;θ)g(w|pl;θ(q))|pl;θ(q)(39c)≤log∫g(w|pl;θ)g(w|pl;θ(q))pl(w)g(w|pl;θ(q))dw(39d)≤log∫g(w|pl;θ)pl(w)dw(39e)≤log∫p(w|pl;θ)dw=0,

where the inequality (39b) is obtained from Jensen’s inequality.

As we can see, the first difference in Equation (38) is nonnegative, while the second one is no larger than zero. Thus, it is easy to get $\log L\left(\theta^{\left(q+1\right)};pl\right)\geq \log L\left(\theta^{\left(q\right)};pl\right)$, and consequently, we have the inequality (32). □

**Corollary** **2.** *When the contour function pl(w) degrades to the categorical mass function, the probability function $p(w|pl;\theta^{\left(q\right)})$ degrades to the conditional probability function $P(\cdot |y,\theta^{\left(q\right)})$ in EM.*

### 3.3. The Estimation Methodology for a Mixed Exponential Distribution

The likelihood function based on the complete data *w* for Type-II progressively censored data from MED is:

(40) $$\begin{array}{cc} L(\pi ,\lambda ;w)\; & =\;\prod_{i=1}^{N}\left[\prod_{k=1}^{m}{\left(\pi_{k}\lambda_{k}e^{-\lambda_{k}t_{i}}\right)}^{b_{ik}^{\left(1\right)}}\cdot \prod_{j=1}^{R_{i}}\prod_{k=1}^{m}{\left(\pi_{k}\lambda_{k}e^{-\lambda_{k}z_{ij}}\right)}^{b_{ijk}^{\left(2\right)}}\right]. \end{array}$$

The log-likelihood is:

(41) $$\begin{array}{cc} log\;L(\pi ,\lambda ;w)\; & =\;\sum_{i=1}^{N}\left[\sum_{k=1}^{m}b_{ik}^{\left(1\right)}\left(log\pi_{k}+log\lambda_{k}-\lambda_{k}t_{i}\right)+\sum_{j=1}^{R_{i}}\sum_{k=1}^{m}b_{ijk}^{\left(2\right)}\left(log\pi_{k}+log\lambda_{k}-\lambda_{k}z_{ij}\right)\right]. \end{array}$$

As before, let θ=(π,λ) denote the unknown parameters of the MED model. Given the initial parameter $\theta^{\left(0\right)}=\left(\pi^{\left(0\right)},\lambda^{\left(0\right)}\right)$, the alternative iterations of the E2M algorithm are described in the following.

- E-step: Let the parameters at the $q_{th}$ iteration be $\theta^{\left(q\right)}=\left(\pi^{\left(q\right)},\lambda^{\left(q\right)}\right)$. The expectation of logL(π,λ;w) with respect to $p(w|pl;\theta^{\left(q\right)})$ (*Q* function) can be defined as:
  (42a)Q(θ,θ(q))=E[logL(π,λ;w)|pl;θ(q)]=∑i=1N{∑k=1m(logπk+logλk−λkti)E[bik(1)|pl;θ(q)]+(42b)∑j=1Ri∑k=1m(logπk+logλk)E[bijk(2)|pl;θ(q)]−λkE[bijk(2)zij|pl;θ(q)]}.
  From Equation (13), we known:
  (43a)p(xi,bik(1)=1|pl;θ(q))=πk(q)fkti;λk(q)plti,k(B)pl(X)ti∑l=1mπl(q)∫xiflxi;λk(q)plxi,l(B)pl(X)xidxi(43b)=πk(q)fkti;λk(q)plti,k(B)pl(X)ti∑l=1mπl(q)flti;λk(q)plti,l(B).
  As $b_{ik}^{\left(1\right)}$ is a Boolean variable, it is easy to get:
  (44) $$E\left[b_{ik}^{\left(1\right)}|pl;\theta^{\left(q\right)}\right]=P\left(b_{ik}^{\left(1\right)}=1|pl;\theta^{\left(q\right)}\right)=\frac{\pi_{k}^{\left(q\right)}f_{k}\left(t_{i};\lambda_{k}^{\left(q\right)}\right)pl_{t_{i},k}^{\left(B\right)}}{\sum_{l=1}^{m}\pi_{l}^{\left(q\right)}f_{l}\left(t_{i};\lambda_{k}^{\left(q\right)}\right)pl_{t_{i},l}^{\left(B\right)}}≜a_{ik}^{\left(q\right)}.$$
  Similarity, we can get:
  (45) $$p\left(z_{ij},b_{ijk}^{\left(2\right)}=1|pl;\theta^{\left(q\right)}\right)\;=\;\frac{\pi_{k}^{\left(q\right)}f_{k}\left(z_{ij};\lambda_{k}^{\left(q\right)}\right)pl_{z_{ij},k}^{\left(B\right)}pl^{\left(X\right)}\left(z_{ij}\right)}{\sum_{l=1}^{m}\pi_{l}^{\left(q\right)}s_{l}\left(z_{ij};\lambda_{k}^{\left(q\right)}\right)pl_{z_{ij},l}^{\left(B\right)}}.$$
  (46) $$E\left[b_{ijk}^{\left(2\right)}|pl;\theta^{\left(q\right)}\right]=P\left(b_{ijk}^{\left(2\right)}=1|pl;\theta^{\left(q\right)}\right)=\frac{\pi_{k}^{\left(q\right)}s_{k}\left(t_{i};\lambda_{k}^{\left(q\right)}\right)pl_{z_{ij},k}^{\left(B\right)}}{\sum_{l=1}^{m}\pi_{l}^{\left(q\right)}s_{l}\left(z_{ij};\lambda_{l}^{\left(q\right)}\right)pl_{z_{ij},l}^{\left(B\right)}}≜e_{ijk}^{\left(q\right)}.$$
  For the expectation of $b_{ijk}^{\left(2\right)}z_{ij}$, we can get:
  (47) $$E\left[b_{ijk}^{\left(2\right)}z_{ij}|pl;\theta^{\left(q\right)}\right]\;=\;E\left[z_{ij}|b_{ijk}^{\left(2\right)}=1,pl;\theta^{\left(q\right)}\right]P\left(b_{ijk}^{\left(2\right)}=1|pl;\theta^{\left(q\right)}\right),$$
  where $P(b_{ijk}^{\left(2\right)}=1|pl;\theta^{\left(q\right)})$ can be obtained by Equation (46). From Equations (45) and (46), we can get:   
  (48) $$p\left(z_{ij}|b_{ijk}^{\left(2\right)}=1,pl;\theta^{\left(q\right)}\right)=\frac{p(z_{ij},b_{ijk}^{\left(2\right)}=1|pl;\theta^{\left(q\right)})}{P(b_{ijk}^{\left(2\right)}=1|pl;\theta^{\left(q\right)})}=\frac{f_{k}\left(z_{ij};\lambda_{k}^{\left(q\right)}\right)pl^{\left(X\right)}\left(z_{ij}\right)}{s_{k}\left(t_{i};\lambda_{k}^{\left(q\right)}\right)}.$$
  As $z_{ij}\in \left[t_{i},+\infty \right)$, we can get:
  (49) $$E\left[z_{ij}|b_{ijk}^{\left(2\right)}=1,pl;\theta^{\left(q\right)}\right]=\int_{t_{i}}^{\infty }z_{ij}\frac{f_{k}\left(z_{ij};\lambda_{k}^{\left(q\right)}\right)}{s_{k}\left(t_{i};\lambda_{k}^{\left(q\right)}\right)}dz_{ij}≜\xi_{ik}^{\left(q\right)}.$$
  Therefore,
  (50) $$E\left[b_{ijk}^{\left(2\right)}z_{ij}|pl;\theta^{\left(q\right)}\right]=\xi_{ik}^{\left(q\right)}e_{ijk}^{\left(q\right)}.$$
  Substituting Equations (44), (46), and (50) into Equation (42b), we can get the *Q* function in the $q_{th}$ iteration:
  (51) $$Q\left(\theta ,\theta^{\left(q\right)}\right)\;=\;\sum_{i=1}^{N}\left[\sum_{k=1}^{m}\left(log\pi_{k}+log\lambda_{k}-\lambda_{k}t_{i}\right)a_{ik}^{\left(q\right)}+\sum_{j=1}^{R_{i}}\sum_{k=1}^{m}\left[\left(log\pi_{k}+log\lambda_{k}\right)e_{ijk}^{\left(q\right)}-\lambda_{k}\xi_{ik}^{\left(q\right)}e_{ijk}^{\left(q\right)}\right]\right],$$
  where $\theta =\left(\pi ,\lambda \right),\pi =\left(\pi_{1},\;\cdots ,\;\pi_{m}\right),\lambda =\left(\lambda_{1},\;\cdots ,\;\lambda_{m}\right)$, and $\sum_{k=1}^{m}\pi_{k}=1$.

**Corollary** **3.** *If there is no uncertain information in the experiment, i.e., $pl_{x_{i},k}=1$, the conditional expectations defined in Equations (44), (46), and (50) are the same as those in the conventional EM algorithm:*

$$\begin{array}{c} E\left[b_{ik}^{\left(1\right)}|pl;\theta^{\left(q\right)}\right]=\frac{\pi_{k}^{\left(q\right)}f_{k}\left(t_{i};\lambda_{k}^{\left(q\right)}\right)}{\sum_{l=1}^{m}\pi_{l}^{\left(q\right)}f_{l}\left(t_{i};\lambda_{l}^{\left(q\right)}\right)},\\ E\left[b_{ijk}^{\left(2\right)}|pl;\theta^{\left(q\right)}\right]=\frac{\pi_{k}^{\left(q\right)}s_{k}\left(t_{i};\lambda_{k}^{\left(q\right)}\right)}{\sum_{l=1}^{m}\pi_{l}^{\left(q\right)}s_{l}\left(t_{i};\lambda_{l}^{\left(q\right)}\right)},\\ E\left[b_{ijk}^{\left(2\right)}z_{ij}|pl;\theta^{\left(q\right)}\right]=\frac{\pi_{k}^{\left(q\right)}\int_{t_{i}}^{\infty }z_{ij}f_{k}\left(z_{ij};\lambda_{k}^{\left(q\right)}\right)dz_{ij}}{\sum_{l=1}^{m}\pi_{l}^{\left(q\right)}s_{l}\left(t_{i};\lambda_{l}^{\left(q\right)}\right)}. \end{array}$$

- M-step: In order to get the updated estimates, we have to maximize the $Q(\theta ,\theta^{\left(q\right)})$ function with respect to parameters π and λ. The Lagrangian method can be adopted to consider the constraint $\sum_{k=1}^{m}\pi_{k}=1$. Let:
  $$L\left(\theta ,\eta \right)\;=\;Q\left(\theta ,\theta^{\left(q\right)}\right)+\eta \left(\sum_{k=1}^{m}\pi_{k}-1\right).$$
  We can get:
  (52) $$\begin{array}{c} \frac{\partial L(\theta ,\eta )}{\partial \pi_{k}}=\frac{A_{k}^{\left(q\right)}}{\pi_{k}}+\eta =0, \end{array}$$
  (53) $$\begin{array}{c} \frac{\partial L(\theta ,\eta )}{\partial \lambda_{k}}=\frac{A_{k}^{\left(q\right)}}{\lambda_{k}}-C_{k}^{\left(q\right)}-\sum_{i=1}^{N}t_{i}a_{ik}^{\left(q\right)}=0, \end{array}$$
  (54) $$\begin{array}{c} \frac{\partial L(\theta ,\eta )}{\partial \eta }=\sum_{k=1}^{m}\pi_{k}-1=0, \end{array}$$
  where
  (55) $$\begin{array}{c} A_{k}^{\left(q\right)}\;=\;\sum_{i=1}^{N}a_{ik}^{\left(q\right)}+\sum_{i=1}^{N}\sum_{j=1}^{R_{i}}e_{ijk}^{\left(q\right)}, \end{array}$$
  (56) $$\begin{array}{c} C_{k}^{\left(q\right)}\;=\;\sum_{i=1}^{N}\sum_{j=1}^{R_{i}}\xi_{ik}^{\left(q\right)}e_{ijk}^{\left(q\right)}. \end{array}$$
  From Equations (52) and (54), we know:
  $$\eta =-\sum_{k=1}^{m}A_{k}^{\left(q\right)}.$$
  Thus, it is easy to get:
  (57) $$\begin{array}{c} \pi_{k}^{\left(q+1\right)}=\frac{A_{k}^{\left(q\right)}}{\sum_{k=1}^{m}A_{k}^{\left(q\right)}},k=1,\;\cdots ,\;m, \end{array}$$
  (58) $$\begin{array}{c} \lambda_{k}^{\left(q+1\right)}=\frac{A_{k}^{\left(q\right)}}{C_{k}^{\left(q\right)}+\sum_{i=1}^{N}t_{i}a_{ik}^{\left(q\right)}},k=1,\;\cdots ,\;m. \end{array}$$
  where $A_{k}^{\left(q\right)}$ and $C_{k}^{\left(q\right)}$ are defined in Equations (55) and (56), respectively.

The E-step and M-step will be repeated iteratively until:

(59) $$\sqrt{\sum_{i=1}^{m}{\left(\pi_{i}^{\left(q+1\right)}-\pi_{i}^{\left(q\right)}\right)}^{2}}\leq \epsilon$$

and:

(60) $$\sqrt{\sum_{i=1}^{m}{\left(\lambda_{i}^{\left(q+1\right)}-\lambda_{i}^{\left(q\right)}\right)}^{2}}\leq \epsilon ,$$

where ϵ is a given small positive constant. Suppose that the algorithm stops at the $l_{th}$ step, then $\theta^{\left(l\right)}=\left(\pi^{\left(l\right)},\lambda^{\left(l\right)}\right)$ are the MELEs for the parameters. The whole process of the estimation is summarized in Algorithm 1.
**Algorithm 1:** The evidential estimation approach for uncertain MED under progressive censoring.**Input:** The observed failures $\left(t_{1},\;\cdots ,\;t_{N}\right)$ and the censoring scheme $\left(R_{1},\;\cdots ,\;R_{N}\right)$.   **Initialization:**  (1)Let q=0  (2)Initialize the parameters $\theta^{\left(q\right)}=\left(\pi^{\left(q\right)},\lambda^{\left(q\right)}\right)$**repeat**  (1)E-step: Calculate the *Q* function based on the current parameters $Q(\theta ,\theta^{\left(q\right)})$.  (2)M-step: Maximize $Q(\theta ,\theta^{\left(q\right)})$ with respect to θ and update the parameters $\theta^{\left(q+1\right)}$.  (3)q=q+1**until** Both Equations (59) and (60) hold true.   **Output:** The MELEs of the parameters.

## 4. Data Analysis

In this section, a real dataset of the air conditioning system of an airplane is considered to illustrate the practical application of the proposed evidential estimation model. The group of failure times of the system is given as follows:

1,3,5,7,11,11,11,12,14,14,14,16,16,20,21,23,42,47,52,62,71,71,87,90,95,120,120,225,246,261.

This dataset was analyzed by different authors using various models [43,44]. In [19], the authors found that the MED model with two components (m=2) provided a good fit to the given failure data. The fitted probability density function can be obtained by the maximal likelihood estimations:

$$\hat{f}\left(x\right)={\hat{\pi }}_{1}{\hat{\lambda }}_{1}e^{-{\hat{\lambda }}_{1}x}+{\hat{\pi }}_{2}{\hat{\lambda }}_{2}e^{-{\hat{\lambda }}_{2}x},x\geq 0,$$

with ${\hat{\pi }}_{1}=0.3459,{\hat{\pi }}_{2}=0.6541,{\hat{\lambda }}_{1}=0.0647,{\hat{\lambda }}_{2}=0.0121$. If we do not have any information about the sub-class labels of each sample, the MLEs of the above MED can be obtained using the EM algorithm [19]:

$${\hat{\pi }}_{1}^{\mathrm{MLE}}=0.1136,{\hat{\pi }}_{2}^{\mathrm{MLE}}=0.8864,{\hat{\lambda }}_{1}^{\mathrm{MLE}}=0.1091,{\hat{\lambda }}_{2}^{\mathrm{MLE}}=0.0161.$$

Suppose we have some uncertain information about the sub-class labels. This type of knowledge can be obtained by experts or from historical data. Here, we can use the evidential *c*-means (ECM) clustering algorithm [31] to obtain the contour function for each sample. From the given failures, it is easy to see that they can be divided into two groups. The credal partition found by ECM can provide us with the plausibility of the data belonging to the sub-groups. In ECM, let C=2, and the other parameters are set as the default. The plausibility matrix describing the partial information about the sub-class label of each sample is illustrated in Table 1.

The maximum likelihood estimations in the sense of evidential likelihood (denoted by MELE) that can integrate the uncertain label information can be obtained:

$${\hat{\pi }}_{1}^{\mathrm{MELE}}=0.3433,{\hat{\pi }}_{2}^{\mathrm{MELE}}=0.6567,{\hat{\lambda }}_{1}^{\mathrm{MELE}}=0.0651,{\hat{\lambda }}_{2}^{\mathrm{MELE}}=0.0121.$$

We can see that the estimation results by the proposed estimation model are closer to the estimations by the complete data.

As we know, in the traditional EM algorithm, the solution depends on the initial values adopted. In order to show the influences of the initial values for the proposed estimation model, we will try with different initialization strategies. For parameter π, we evoke the estimation model with:

$$\pi_{1}^{\left(0\right)}=\left|0.5+N\left(0,0.1\right)\right|,\pi_{2}^{\left(0\right)}=1-\pi_{1}^{\left(0\right)},$$

while for λ, we set:

$$\lambda_{1}^{\left(0\right)}=|0.2+N\left(0,0.1\right)|,\lambda_{2}^{\left(0\right)}=\left|0.8+N\left(0,0.1\right)\right|,$$

where |x| is the absolute value of *x* and N(0,0.1) denotes a random number from the normal distribution with $\mu =0,\sigma^{2}=0.1$. The E2M algorithm and EM algorithm are both repeated 100 times. The mean value and the standard variances of the estimations are reported, which is illustrated in Table 2.

It is easy to see that the standard variation values of MELEs are smaller than those of MLEs. We can conclude that the use of the uncertain information can reduce the influence of the initializations in the estimation model. Many initialization methods have been studied for the EM algorithm, which is worth learning and a reference for the proposed estimation model. However, in this paper, we do not focus on the proper initialization method and leave it for our future research.

## 5. Simulations

Monte Carlo simulations are conducted in this section to show the performance of the proposed method. Suppose that $X_{1},X_{2},\;\cdots ,\;X_{n}$ are *n* independent variables from the mixed exponential distribution in Equation (2). Here, we consider the model with two mixture components (m=2) under the Type-II progressive censoring scheme. The parameters to be estimated are $\pi_{1},\pi_{2},\lambda_{1},\lambda_{2}$ with $\pi_{1}+\pi_{2}=1$.

To simulate uncertainty on class labels, the original generated data are processed as follows. For the $i_{th}$ observed failure, an error probability $q_{i}$ is drawn randomly from a beta distribution with mean ρ and standard deviation σ. In real applications, $q_{i}$ can be obtained by experts or from historical data. With probability $q_{i}$, the label of $z_{i}$ is replaced by a completely value of ${\tilde{z}}_{i}$ (with a uniform distribution over all the class labels). The contour function on class labels can be defined as:

(61) $$pl_{x_{i},k}^{\left(B\right)}=P\left(b_{ik}=1|b_{ik}^{*}\right)=\left\{\begin{array}{cc} \frac{q_{i}}{m}, & b_{ik}^{*}=0,\\ \frac{q_{i}}{m}+1-q_{i}, & b_{ik}^{*}=1, \end{array}\right.\;\;i=1,\;\cdots ,\;n;\;k=1,\;\cdots ,\;m.$$

If there is no uncertainty, i.e., $pl_{x_{i},k}=1$, the evidential likelihood degrades to the traditional likelihood.

Let $\pi_{1}=0.3,\pi_{2}=0.7;\lambda_{1}=0.6,\lambda_{2}=0.1$. Set the initial value of the parameters as:

$$\pi_{1}^{\left(0\right)}=0.5,\pi_{2}^{\left(0\right)}=0.5,\lambda_{1}^{\left(0\right)}=1,\lambda_{2}^{\left(0\right)}=0.5.$$

In this experiment, we set the number of units placed on the life-test to be n=20,100,200,400. We consider different censoring proportions for a given sample size. Let N=n×(0.1,0.2,0.3). For the parameters to model the uncertain label information, we set ρ=0.2,σ=0.2. Three censoring schemes are considered:

$$\begin{array}{l} \mathrm{Scheme}\;1:\;R_{1}=\cdots ,R_{N-1}=0,R_{N}=n-N.\\ \mathrm{Scheme}\;2:\;R_{1}=n-N,R_{2}=\cdots ,R_{N}=0.\\ \mathrm{Scheme}\;3:\;R_{1}=R_{2}=\cdots =R_{N-1}=1,R_{N}=n-2*N+1. \end{array}$$

Given the sample size *n* and the censoring scheme, the E2M algorithm will be repeated s=100 times. The bias and mean squared error (MSE) values of the estimators are reported. The results are illustrated in Table 3, Table 4, Table 5, Table 6, Table 7 and Table 8.

From the tables, we can see that, with the increasing size of *n* and *N*, for the estimators obtained both by the proposed evidential estimation model and by the EM algorithm, the values of the bias and MSE decrease in most cases. This is consistent with our common sense about the maximum likelihood estimations. For different censoring schemes, it is obvious that the estimation efficiency is highly related to the proportion of the censored samples. When the number of units placed in the life-test is fixed, with the increasing number of censored data, the values of MSE increase.

Overall, in most cases, the biases and MSEs of the MELEs by the proposed evidential estimation model are smaller than those of MLEs by the EM algorithm, which can be attributed to the use of the uncertain prior information about the sub-class labels. When the proportion of the censored data is large, the MELEs obtained by the proposed estimation model are significantly better.

## 6. Conclusions

In this paper, a method for estimating the parameters of a mixed distribution model under Type-II progressive censoring was introduced. The main advantage of the proposed formalism is that it can combine uncertainty captured by the imperfect observation process due to censoring and the partial knowledge about the sub-class information in the mixture model. These two types of uncertain information can be represented in a united frame by the use of belief functions, and then, the evidential likelihood is derived, which can be seen as a generalized likelihood criterion. It can be maximized using the evidential EM algorithm. As an illustration, the method was applied to the MED model. A real dataset was considered, and some Monte Carlo simulations were carried out to demonstrate the performance of the resulting estimates. The results show that the estimations by the proposed method gain better estimation efficiency, which can be attributed to the effective integration of the uncertain information in the model.

More generally, the evidential estimation approach introduced in this paper is applicable to any other mixed distributions under different censoring schemes, where data uncertainty in the parametric statistical model arises from the imperfect observation process and reliability engineering applications such as the life data with multiple failure causes.

## Figures and Tables

**Table 1:** The plausibility matrix for the data.

ID	${\mathrm{pl}}_{x_{i},1}^{\left(B\right)}$	${\mathrm{pl}}_{x_{i},2}^{\left(B\right)}$	ID	${\mathrm{pl}}_{x_{i},1}^{\left(B\right)}$	${\mathrm{pl}}_{x_{i},2}^{\left(B\right)}$
1	0.9971	0.0080	16	0.9976	0.0076
2	0.9980	0.0057	17	0.9775	0.0858
3	0.9987	0.0038	18	0.9687	0.1264
4	0.9992	0.0022	19	0.9590	0.1770
5	0.9999	0.0003	20	0.9388	0.3094
6	0.9999	0.0003	21	0.9247	0.4584
7	0.9999	0.0003	22	0.9247	0.4584
8	1.0000	0.0001	23	0.9266	0.7394
9	1.0000	0.0001	24	0.9319	0.7860
10	1.0000	0.0001	25	0.9435	0.8549
11	1.0000	0.0001	26	1.0000	1.0000
12	0.9998	0.0006	27	1.0000	1.0000
13	0.9998	0.0006	28	0.0001	1.0000
14	0.9989	0.0035	29	0.0189	0.9930
15	0.9985	0.0047	30	0.0474	0.9813

**Table 2:** The mean and standard variance (sd) values of the estimations with different initializations. MELE, maximal evidential likelihood estimate.

	MLE		MELE	
**Parameter**	**Mean**	**sd**	**Mean**	**sd**
$\lambda_{1}$	0.0898	0.0101	0.0598	0.0083
$\lambda_{2}$	0.0177	0.0305	0.0277	0.0045
$\pi_{1}$	0.1931	0.1094	0.0931	0.0182
$\pi_{2}$	0.8069	0.1094	0.4069	0.0154

**Table 3:** The statistical properties of the estimators of $\lambda_{1}$ and $\lambda_{2}$ with Scheme 1. ${\hat{\lambda }}_{i}^{\mathrm{MELE}}$ and ${\hat{\lambda }}_{i}^{\mathrm{MLE}}$ are the MELE by the proposed estimation method and the MLE by the traditional EM algorithm, respectively.

		Bias				MSE			
n	N	${\hat{\lambda }}_{1}^{\mathrm{MLE}}$	${\hat{\lambda }}_{1}^{\mathrm{MELE}}$	${\hat{\lambda }}_{2}^{\mathrm{MLE}}$	${\hat{\lambda }}_{2}^{\mathrm{MELE}}$	${\hat{\lambda }}_{1}^{\mathrm{MLE}}$	${\hat{\lambda }}_{1}^{\mathrm{MELE}}$	${\hat{\lambda }}_{2}^{\mathrm{MLE}}$	${\hat{\lambda }}_{2}^{\mathrm{MELE}}$
20	2	0.3940	0.2279	0.3193	0.1421	5.4890	3.6382	0.0990	0.0808
20	4	0.9357	0.7783	0.2508	0.1373	4.4376	2.6257	0.0933	0.0764
20	6	0.1281	1.0112	0.1934	0.1189	5.9467	2.6358	0.0858	0.0683
100	10	0.7666	0.2661	0.2825	0.1249	3.1009	0.0790	0.0970	0.0642
100	20	0.4059	0.2158	0.2504	0.1509	1.6919	0.3333	0.0823	0.0652
100	30	0.2601	0.1422	0.1860	0.1427	0.6354	0.1728	0.0865	0.0657
200	20	0.6175	0.2911	0.2775	0.1167	7.9779	0.7749	0.1008	0.0645
200	40	0.2238	0.2470	0.2451	0.1639	1.0183	0.5144	0.0747	0.0604
200	60	0.4149	0.1986	0.1992	0.1454	3.9797	0.3526	0.0810	0.0665
400	40	0.9731	0.3065	0.2739	0.1268	7.4599	0.0686	0.0936	0.0635
400	80	0.7262	0.3163	0.2461	0.1242	9.7900	0.0716	0.0820	0.0540
400	120	0.2421	0.1978	0.2037	0.1219	0.3790	0.2534	0.0768	0.0455

**Table 4:** The statistical properties of the estimators of $\pi_{1}$ and $\pi_{2}$ with Scheme 1. ${\hat{\pi }}_{i}^{\mathrm{MELE}}$ and ${\hat{\pi }}_{i}^{\mathrm{MLE}}$ are the MELE by the proposed estimation method and the MLE by the traditional EM algorithm, respectively.

		Bias				MSE			
n	N	${\hat{\pi }}_{1}^{\mathrm{MLE}}$	${\hat{\pi }}_{1}^{\mathrm{MELE}}$	${\hat{\pi }}_{2}^{\mathrm{MLE}}$	${\hat{\pi }}_{2}^{\mathrm{MELE}}$	${\hat{\pi }}_{1}^{\mathrm{MLE}}$	${\hat{\pi }}_{1}^{\mathrm{MELE}}$	${\hat{\pi }}_{2}^{\mathrm{MLE}}$	${\hat{\pi }}_{2}^{\mathrm{MELE}}$
20	2	0.0508	0.0892	0.0579	0.1193	0.3963	0.2967	0.1314	0.0596
20	4	0.1200	0.0392	0.0627	0.0508	0.1669	0.1140	0.1275	0.0752
20	6	0.1178	0.0739	0.0811	0.0066	0.1644	0.1364	0.1272	0.0869
100	10	0.1316	0.0589	0.0751	0.0825	0.1578	0.1116	0.1339	0.0546
100	20	0.1471	0.0810	0.0491	0.0504	0.1643	0.1215	0.1122	0.0649
100	30	0.1695	0.1146	0.0573	0.0140	0.1795	0.1416	0.1180	0.0861
200	20	0.1420	0.0718	0.0833	0.0775	0.1596	0.1150	0.1402	0.0561
200	40	0.1503	0.0917	0.0361	0.0451	0.1674	0.1281	0.1014	0.0626
200	60	0.1744	0.1190	0.0546	0.0008	0.1820	0.1430	0.1121	0.0819
400	40	0.1529	0.0801	0.0732	0.0739	0.1685	0.1189	0.1304	0.0563
400	80	0.1629	0.0946	0.0558	0.0461	0.1751	0.1260	0.1140	0.0649
400	120	0.1799	0.1170	0.0581	0.0037	0.1860	0.1405	0.1097	0.0794

**Table 5:** The statistical properties of the estimators of $\lambda_{1}$ and $\lambda_{2}$ with Scheme 2. ${\hat{\lambda }}_{i}^{\mathrm{MELE}}$ and ${\hat{\lambda }}_{i}^{\mathrm{MLE}}$ are the MELE by the proposed estimation method and the MLE by the traditional EM algorithm, respectively.

		Bias				MSE			
n	N	${\hat{\lambda }}_{1}^{\mathrm{MLE}}$	${\hat{\lambda }}_{1}^{\mathrm{MELE}}$	${\hat{\lambda }}_{2}^{\mathrm{MLE}}$	${\hat{\lambda }}_{2}^{\mathrm{MELE}}$	${\hat{\lambda }}_{1}^{\mathrm{MLE}}$	${\hat{\lambda }}_{1}^{\mathrm{MELE}}$	${\hat{\lambda }}_{2}^{\mathrm{MLE}}$	${\hat{\lambda }}_{2}^{\mathrm{MELE}}$
20	2	9.1642	10.1750	0.1960	0.1354	4.8531	9.1105	0.0942	0.1050
20	4	12.2537	13.2526	0.2083	0.1614	5.6519	5.0538	0.0905	0.0959
20	6	4.0073	4.3486	0.2546	0.1650	1.3179	4.2556	0.1318	0.0993
100	10	12.9948	9.4179	0.1790	0.1272	7.8862	9.7824	0.0925	0.1078
100	20	1.2818	1.3895	0.1991	0.1259	11.6120	3.5432	0.1077	0.1003
100	30	0.4817	0.7400	0.2286	0.1477	1.9537	5.4708	0.1082	0.0890
200	20	1.9440	0.9701	0.2530	0.1425	2.9915	1.5640	0.1163	0.1022
200	40	0.3792	0.5135	0.2234	0.1697	1.6746	4.1021	0.0834	0.0781
200	60	0.2999	1.2155	0.2286	0.1742	1.1539	1.5056	0.0962	0.0792
400	40	2.6339	1.3680	0.1942	0.1198	1.1806	1.7962	0.0848	0.0982
400	80	4.7986	0.6004	0.2031	0.1395	1.3783	0.5436	0.0840	0.0810
400	120	0.2702	0.0976	0.2199	0.1410	0.6702	0.4962	0.0924	0.0876

**Table 6:** The statistical properties of the estimators of $\pi_{1}$ and $\pi_{2}$ with Scheme 2. ${\hat{\pi }}_{i}^{\mathrm{MELE}}$ and ${\hat{\pi }}_{i}^{\mathrm{MLE}}$ are the MELE by the proposed estimation method and the MLE by the traditional EM algorithm, respectively.

		Bias				MSE			
n	N	${\hat{\pi }}_{1}^{\mathrm{MLE}}$	${\hat{\pi }}_{1}^{\mathrm{MELE}}$	${\hat{\pi }}_{2}^{\mathrm{MLE}}$	${\hat{\pi }}_{2}^{\mathrm{MELE}}$	${\hat{\pi }}_{1}^{\mathrm{MLE}}$	${\hat{\pi }}_{1}^{\mathrm{MELE}}$	${\hat{\pi }}_{2}^{\mathrm{MLE}}$	${\hat{\pi }}_{2}^{\mathrm{MELE}}$
20	2	0.0308	0.0205	0.0646	0.0040	0.2471	0.2576	0.1382	0.1103
20	4	0.0765	0.0688	0.0386	0.0083	0.2212	0.1841	0.1215	0.1036
20	6	0.1701	0.1613	0.0350	0.0546	0.1786	0.1867	0.1235	0.1256
100	10	0.1830	0.1791	0.0728	0.0210	0.1831	0.1849	0.1425	0.1148
100	20	0.1936	0.1878	0.0741	0.0009	0.1916	0.1972	0.1369	0.1215
100	30	0.1968	0.1897	0.0523	0.0286	0.1915	0.1977	0.1186	0.1129
200	20	0.1976	0.1832	0.0575	0.0530	0.1893	0.1999	0.1330	0.1140
200	40	0.1974	0.1926	0.0303	0.0234	0.1941	0.1976	0.1028	0.0929
200	60	0.1979	0.1934	0.0258	0.0286	0.1943	0.1986	0.1030	0.1023
400	40	0.2011	0.1929	0.0802	0.0058	0.1945	0.2008	0.1365	0.1017
400	80	0.1965	0.1905	0.0605	0.0031	0.1926	0.1978	0.1149	0.0983
400	120	0.2001	0.1931	0.0590	0.0199	0.1956	0.2020	0.1195	0.0981

**Table 7:** The statistical properties of the estimators of $\lambda_{1}$ and $\lambda_{2}$ with Scheme 3. ${\hat{\lambda }}_{i}^{\mathrm{MELE}}$ and ${\hat{\lambda }}_{i}^{\mathrm{MLE}}$ are the MELE by the proposed estimation method and the MLE by the traditional EM algorithm, respectively.

		Bias				MSE			
n	N	${\hat{\lambda }}_{1}^{\mathrm{MLE}}$	${\hat{\lambda }}_{1}^{\mathrm{MELE}}$	${\hat{\lambda }}_{2}^{\mathrm{MLE}}$	${\hat{\lambda }}_{2}^{\mathrm{MELE}}$	${\hat{\lambda }}_{1}^{\mathrm{MLE}}$	${\hat{\lambda }}_{1}^{\mathrm{MELE}}$	${\hat{\lambda }}_{2}^{\mathrm{MLE}}$	${\hat{\lambda }}_{2}^{\mathrm{MELE}}$
20	2	7.9040	0.1744	0.1278	0.1974	8.9819	0.2180	0.1060	0.0689
20	4	3.0177	5.2017	0.1306	0.1556	7.6359	1.3141	0.1031	0.0694
20	6	1.4218	0.5737	0.1603	0.1936	3.1824	1.1476	0.0872	0.0804
100	10	1.7309	0.0356	0.1230	0.1750	4.4582	1.5949	0.0954	0.0651
100	20	0.9920	0.1063	0.1410	0.1343	3.2922	1.3056	0.0891	0.0678
100	30	0.5160	0.4788	0.1254	0.1470	1.6549	2.8739	0.0843	0.0732
200	20	0.8511	0.2909	0.1285	0.1770	4.9667	0.0717	0.0902	0.0638
200	40	0.4303	0.3190	0.1322	0.1393	1.5101	0.0742	0.0873	0.0649
200	60	0.6432	0.4140	0.1272	0.1505	4.1079	2.7520	0.0851	0.0778
400	40	0.4359	0.3001	0.1124	0.1753	1.2242	0.0669	0.0971	0.0630
400	80	0.0547	0.3164	0.1377	0.0423	0.9854	0.1682	0.0896	0.0628
400	120	0.0770	0.0161	0.1391	0.0752	0.9764	0.0856	0.0829	0.0743

**Table 8:** The statistical properties of the estimators of $\pi_{1}$ and $\pi_{2}$ with Scheme 3. ${\hat{\pi }}_{i}^{\mathrm{MELE}}$ and ${\hat{\pi }}_{i}^{\mathrm{MLE}}$ are the MELE by the proposed estimation method and the MLE by the traditional EM algorithm, respectively.

		Bias				MSE			
n	N	${\hat{\pi }}_{1}^{\mathrm{MLE}}$	${\hat{\pi }}_{1}^{\mathrm{MELE}}$	${\hat{\pi }}_{2}^{\mathrm{MLE}}$	${\hat{\pi }}_{2}^{\mathrm{MELE}}$	${\hat{\pi }}_{1}^{\mathrm{MLE}}$	${\hat{\pi }}_{1}^{\mathrm{MELE}}$	${\hat{\pi }}_{2}^{\mathrm{MLE}}$	${\hat{\pi }}_{2}^{\mathrm{MELE}}$
20	2	0.0438	0.0149	0.0722	0.0974	0.2629	0.2063	0.1415	0.0554
20	4	0.0771	0.0028	0.0694	0.0556	0.1643	0.1263	0.1377	0.0686
20	6	0.1432	0.0668	0.0397	0.0064	0.1712	0.1335	0.1140	0.0967
100	10	0.1445	0.0685	0.0770	0.0750	0.1617	0.1120	0.1335	0.0576
100	20	0.1526	0.0947	0.0590	0.0343	0.1692	0.1294	0.1214	0.0722
100	30	0.1806	0.1440	0.0746	0.0530	0.1861	0.1644	0.1221	0.1055
200	20	0.1463	0.0718	0.0715	0.0770	0.1624	0.1153	0.1273	0.0557
200	40	0.1685	0.1000	0.0678	0.0393	0.1793	0.1290	0.1225	0.0677
200	60	0.1771	0.1463	0.0728	0.0495	0.1834	0.1615	0.1220	0.1078
400	40	0.1511	0.0763	0.0876	0.0753	0.1675	0.1182	0.1384	0.0555
400	80	0.1644	0.0977	0.0623	0.0423	0.1782	0.1289	0.1223	0.0652
400	120	0.1718	0.1415	0.0609	0.0248	0.1794	0.1557	0.1156	0.0961
